# Supplementary material for: PhyloQuant approach provides insights into Trypanosoma cruzi evolution using a systems-wide mass spectrometry-based quantitative protein profile
Source: Commun Biol. 2021 Mar 11;4:324. doi: 10.1038/s42003-021-01762-6 (PMC7952728; doi:10.1038/s42003-021-01762-6)
Supplement: Supplementary file 2 — Supplementary Information [file 42003_2021_1762_MOESM2_ESM.pdf]

PhyloQuant approach provides insights into *Trypanosoma cruzi* evolution using a systems-wide mass spectrometry-based quantitative protein profile

Simon Ngao Mule[1], André Guilherme Costa-Martins[1], Livia Rosa-Fernandes[1], Gilberto Santos de Oliveira[1], Carla Monadeli F. Rodrigues[1], Daniel Quina[1], Graziella E. Rosein[2], Marta Maria Geraldes Teixeira[1] and Giuseppe Palmisano[1]*

1 Department of Parasitology, Institute of Biomedical Sciences, University of São Paulo, Brazil

2 Department of Biochemistry, Institute of Chemistry, University of São Paulo, Brazil

* To whom correspondence should be addressed:

Prof. Giuseppe Palmisano, Glycoproteomics Laboratory, Department of Parasitology, ICB,

University of São Paulo, Brazil, Av. Prof. Lineu Prestes, 1374, 05508 900 São Paulo SP Brazil,

Tel: + 55 11 99920 8662, palmisano.gp@gmail.com , palmisano.gp@usp.br

Supplementary Information

Supplementary Fig. 1 and Supplementary Fig. 2 (available in this file)

Supplementary Data 1 – 5 legends. Supplementary Tables are available as separate MS Excel files.

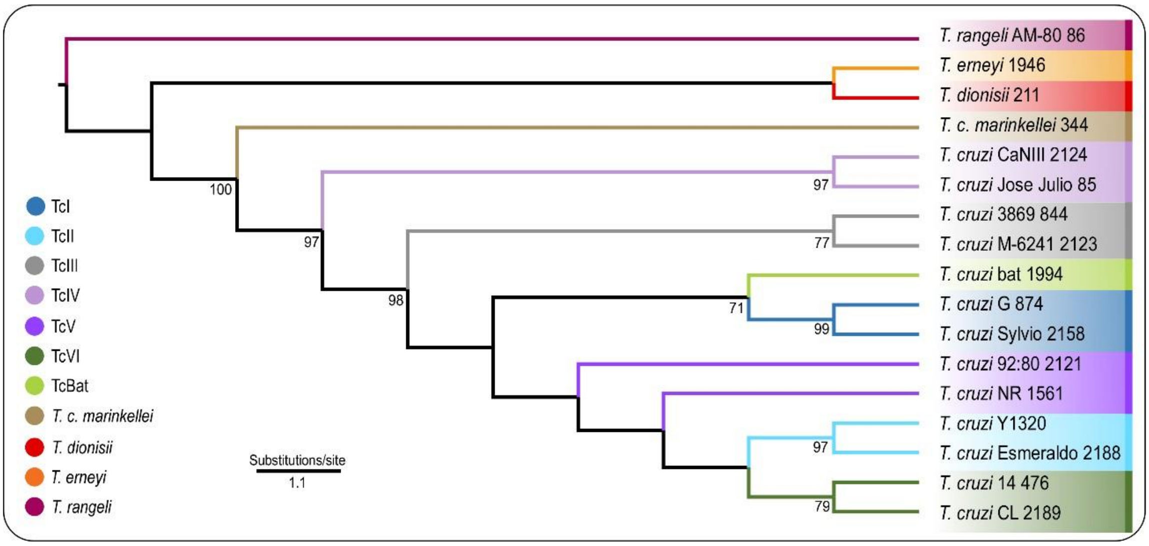

Supplementary Fig. 1: Phylogenetic tree showing clustering of *T. cruzi* DTUs and allied trypanosome species in the *Schizotrypanum* clade constructed using Maximum Parsimony phylogenetic algorithm. The phylogenetic tree is inferred from concatenated SSU rRNA, gGAPDH and HSP70 gene sequences. *T. rangeli* was included as an outgroup to the *Schizotrypanum* clade. Branch support values above 70 % are included.

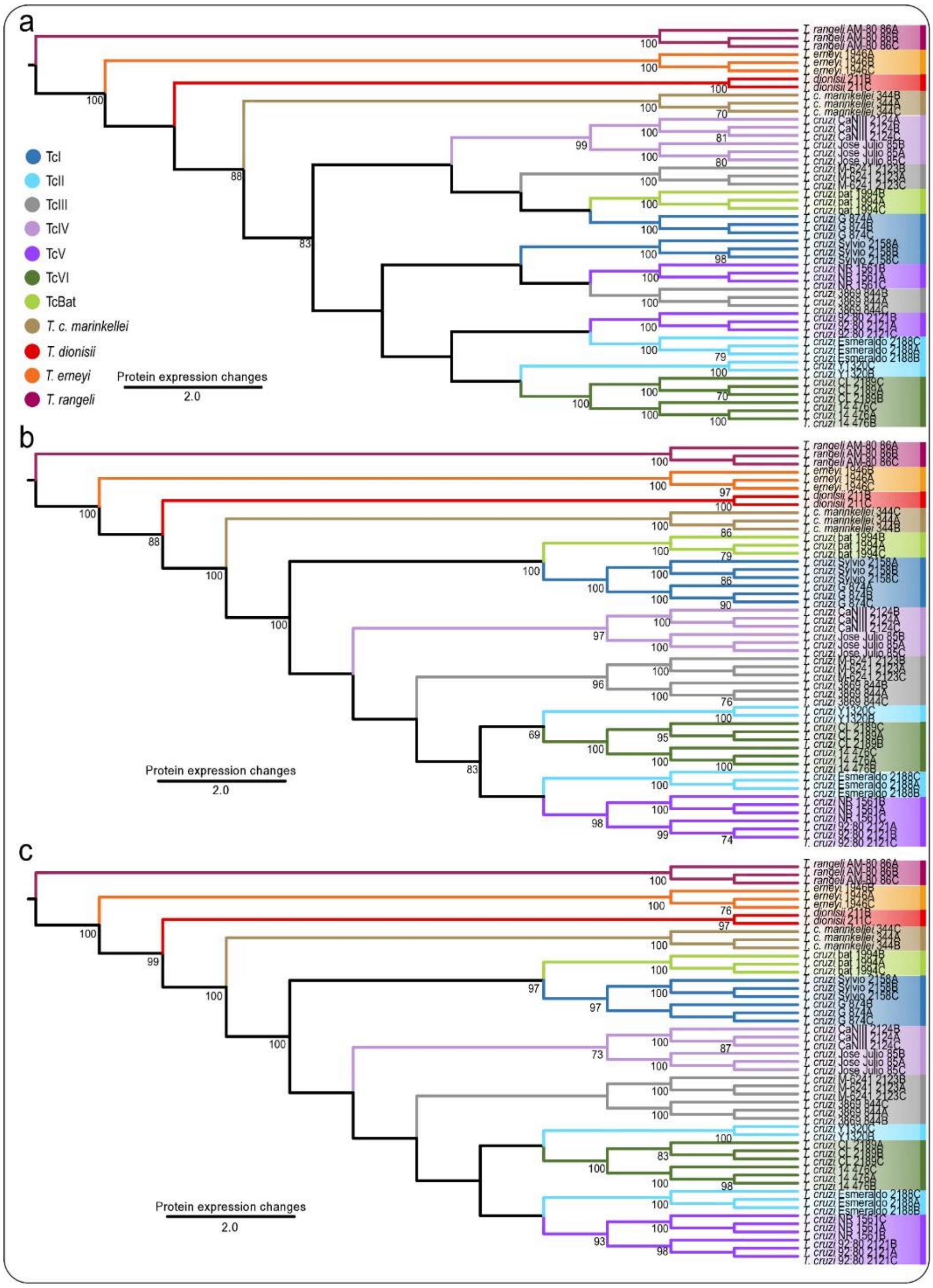

Supplementary Fig. 2: PhyloQuant trees based on total identified and quantified MS features. Phylogenies based on total a) MS1, b) iBAQ, and c) LFQ intensities inferred using the PhyloQuant approach are demonstrated. Branch support values above 70 % are included.

Supplementary Data 1: Quantified MS1 intensities. i) Total quantified MS1 Intensities with charge state 2-7, and, **ii)** Normalized MS1 intensities **iii**) Imputed normalized MS1 intensities identified with 3 valid values in at least 1 condition, **iv**) Statistically significant MS1 values using Benjamini-Hochberg based FDR correction at an FDR < 0.05.

Supplementary Data 2: Identified and quantified proteins based on LFQ intensities. i) Total identified proteins, **ii)** proteins identified and quantified with a minimum of 3 valid values in at least 1 condition, and, **iii)** statistically regulated and imputed proteins based on LFQ intensities using Benjamini-Hochberg-based FDR correction at an FDR < 0.05.

Supplementary Data 3: Identified and Quantified proteins based on iBAQ intensities. i) Total identified iBAQ intensities **ii)** normalized iBAQ intensities, **iii**) IBAQ intensities identified and quantified with a minimum of 3 valid values in at least 1 condition, and, **iv)** statistically regulated and imputed iBAQ intensities using Benjamini-Hochberg-based FDR correction at an FDR < 0.05.

Supplementary Data 4: Synapomorphies mapped for *T. cruzi* clades and closely related trypanosome species based on PhyloQuant inferred from statistically significant LFQ intensities.

Supplementary Data 5: Strain/Species and DTU specific proteins.
