# Supplementary material for: PhyloQuant approach provides insights into Trypanosoma cruzi evolution using a systems-wide mass spectrometry-based quantitative protein profile
Source: Commun Biol. 2021 Mar 11;4:324. doi: 10.1038/s42003-021-01762-6 (PMC7952728; doi:10.1038/s42003-021-01762-6)
Supplement: Supplementary file 3 — Description of Additional Supplementary Files [file 42003_2021_1762_MOESM3_ESM.pdf]

Description of Additional Supplementary Files

File name: Supplementary Data 1.

Description: Quantified MS1 intensities. i) Total quantified MS1 Intensities with charge state 2-7, and, ii) Normalized MS1 intensities iii) Imputed normalized MS1 intensities identified with 3 valid values in at least 1 condition, iv) Statistically significant MS1 values using Benjamini-Hochberg based FDR correction at an FDR < 0.05.

File name: Supplementary Data 2.

Description: Identified and quantified proteins based on LFQ intensities. i) Total identified proteins, ii) proteins identified and quantified with a minimum of 3 valid values in at least 1 condition, and, iii) statistically regulated and imputed proteins based on LFQ intensities using Benjamini-Hochberg-based FDR correction at an FDR < 0.05.

File name: Supplementary Data 3.

Description: Identified and quantified proteins based on iBAQ intensities. i) Total identified iBAQ intensities ii) normalized iBAQ intensities, iii) IBAQ intensities identified and quantified with a minimum of 3 valid values in at least 1 condition, and, iv) statistically regulated and imputed iBAQ intensities using Benjamini-Hochberg-based FDR correction at an FDR < 0.05.

File name: Supplementary Data 4.

Description: Synapomorphies mapped for T. cruzi clades and closely related trypanosome species based on PhyloQuant inferred from statistically significant LFQ intensities.

File name: Supplementary Data 5.

Description: Strain/Species and DTU specific proteins.
