# Supplementary material for: PhyloQuant approach provides insights into Trypanosoma cruzi evolution using a systems-wide mass spectrometry-based quantitative protein profile
Source: Commun Biol. 2021 Mar 11;4:324. doi: 10.1038/s42003-021-01762-6 (PMC7952728; doi:10.1038/s42003-021-01762-6)
Supplement: Supplementary file 9 — Reporting Summary [file 42003_2021_1762_MOESM9_ESM.pdf]

nature research

Corresponding author(s): DBPR COMMSBIO-20-0259A

Last updated by author(s): Sep 5, 2020

Reporting Summary

Nature Research wishes to improve the reproducibility of the work that we publish. This form provides structure for consistency and transparency in reporting. For further information on Nature Research policies, see our Editorial Policies and the Editorial Policy Checklist.

Statistics

For all statistical analyses, confirm that the following items are present in the figure legend, table legend, main text, or Methods section.

n/a	Confirmed	
☐	☒	The exact sample size (*n*) for each experimental group/condition, given as a discrete number and unit of measurement
☐	☒	A statement on whether measurements were taken from distinct samples or whether the same sample was measured repeatedly
☐	☒	The statistical test(s) used AND whether they are one- or two-sided<br>*Only common tests should be described solely by name; describe more complex techniques in the Methods section.*
☒	☐	A description of all covariates tested
☐	☒	A description of any assumptions or corrections, such as tests of normality and adjustment for multiple comparisons
☐	☒	A full description of the statistical parameters including central tendency (e.g. means) or other basic estimates (e.g. regression coefficient) AND variation (e.g. standard deviation) or associated estimates of uncertainty (e.g. confidence intervals)
☐	☒	For null hypothesis testing, the test statistic (e.g. *F*, *t*, *r*) with confidence intervals, effect sizes, degrees of freedom and *P* value noted<br>*Give P values as exact values whenever suitable.*
☒	☐	For Bayesian analysis, information on the choice of priors and Markov chain Monte Carlo settings
☒	☐	For hierarchical and complex designs, identification of the appropriate level for tests and full reporting of outcomes
☒	☐	Estimates of effect sizes (e.g. Cohen's *d*, Pearson's *r*), indicating how they were calculated

Our web collection on statistics for biologists contains articles on many of the points above.

Software and code

Policy information about availability of computer code

Data collection

Nano LC-MS/MS analysis

LC-MS/MS analysis were performed on an EASY-Spray PepMap® 50 cm × 75 um C18 column using an Easy nLC1000 nanoflow system coupled to Orbitrap Fusion Lumos mass spectrometer (Thermo Fischer Scientific, Waltham, MA, USA). The HPLC gradient was 5–25% solvent B (A = 0.1% formic acid; B = 100% ACN, 0.1% formic acid) in 90 min at a flow of 300 nL/min. The most intense precursors selected from the FT MS1 full scan (resolving power 120,000 at m/z 200) were quadrupole-isolated and fragmented by collision-induced dissociation (CID) and detected in the dual-pressure linear ion trap with 30 as normalized collision energy. The MS1 scan range was between 380–1500 m/z, the ion count target was set to 2 × 105, the MS2 ion count target was set to 1 × 104, and the max injection time was 50 ms and 35 ms for MS1 and MS2, respectively. The dynamic exclusion duration was set to 10 s with a 10-ppm tolerance around the selected precursor and its isotopes. The maximum total cycle time was confined to 3s 23. The mass spectrometry data were collected using the Xcalibur software (Thermo Fisher).

Data analysis

Peptide and Protein identification and quantification

LC-MS/MS raw files were analyzed using MaxQuant v1.5.2.8 for identification and label-free quantification (LFQ) of proteins and peptides. Using the following parameters, MS/MS spectra were searched against the combined reference Uniprot proteome databases of T. cruzi CL Brener, T. cruzi Dm 28c, T. cruzi marinkellei B7 and T. rangeli (strains SC58 and AM80) (Released, March, 2020; 50,075 entries) and common contaminants protein database with a mass tolerance level of 4.5 ppm for MS and 0.5 Da for MS/MS.

Evolutionary resolution of Schizotrypanum lineage

Statistically significant MS1 intensities were determined by analysis of variance (ANOVA) with Benjamini-Hochberg-based false discovery rate (FDR) correction at an FDR < 0.05. To infer evolutionary relationships between the trypanosome species based on MS1 intensities, total and statistically regulated MS1 intensities were normalized by Z score transformation (standard deviations from means) in Perseus computational platform. To build character-based matrix for maximum parsimony method, the proteomic matrix was transposed to present operational taxonomic units (OTUs) in rows and the intensities in columns. Subsequently, Z scores were transformed into character states by rounding decimal fractions to the nearest integers, and the negative integers were substituted by corresponding letters. Finally, Tree Analyses using New Technology (TNT)78 version 1.563 was employed to infer evolutionary relationships using maximum parsimony (MP). Branch statistical

supports were obtained as implemented in TNT using 1000 replicates.

For manuscripts utilizing custom algorithms or software that are central to the research but not yet described in published literature, software must be made available to editors and reviewers. We strongly encourage code deposition in a community repository (e.g. GitHub). See the Nature Research guidelines for submitting code & software for further information.

Data

Policy information about availability of data

All manuscripts must include a data availability statement. This statement should provide the following information, where applicable:

- Accession codes, unique identifiers, or web links for publicly available datasets
- A list of figures that have associated raw data
- A description of any restrictions on data availability

All mass spectrometry raw data have been submitted to PRIDE archive, project accession:PXD017228. The sequenced genes have been deposited in GenBank under the accession numbers MW345242, MW345243, MW345244, MW345245, MW345246 and MW325707.

Field-specific reporting

Please select the one below that is the best fit for your research. If you are not sure, read the appropriate sections before making your selection.

☒ Life sciences ☐ Behavioural & social sciences ☐ Ecological, evolutionary & environmental sciences

For a reference copy of the document with all sections, see nature.com/documents/nr-reporting-summary-flat.pdf

Life sciences study design

All studies must disclose on these points even when the disclosure is negative.

Sample size	Sample size determination was not performed. We used three (3) biological replicates for the trypanosome strains/species in this study. Three biological replicates have been used without compromising the statistical power of the tests performed. In two samples (TCC1320 - T. cruzi Y strain) and TCC211 - T. dionisii), two biological replicates were included. Other studies using quantitative proteomics performed on Trypanosomatid cell lines have been based on biological duplicates or triplicates.<br>We have used several statistical tests to access the protein regulation (ANOVA with multiple sample test Benjamini-Hochberg correction), comparison of evolutionary trees (Mantel test) and inference of protein expression during evolution (pearson correlation).
Data exclusions	One replicate for TCC1320 and TCC211 were excluded prior to data analysis because the mass spectrometry runs did not pass the quality control based on number of MS/MS spectra acquired and chromatographic profiles. This exclussion did not interfere with subsequent data analysis because the biological duplicates were used to perform clustering analysis.
Replication	We tested the reproducubility of the correlation between phyloquant masss spectrometry based clustering and phylogenetics using another trypanosomatid genus (Leishmania). T. cruzi marinkellei and T. rangeli specific protens were confirmed by other studies that described these proteins as potential markers.
Randomization	Each T. cruzi strain and closly related trypanosomatid species were genotyped and placed in their correct clades before hand. However, we used randomization using bootstrap values for each clustering calculated using maximum parsimony.
Blinding	Blinding was not relevant for this study because the data analyses performed were unsupervised, such as principal component analysis (PCA) and parsimony based clustering.

Reporting for specific materials, systems and methods

We require information from authors about some types of materials, experimental systems and methods used in many studies. Here, indicate whether each material, system or method listed is relevant to your study. If you are not sure if a list item applies to your research, read the appropriate section before selecting a response.

Materials & experimental systems

n/a	Involved in the study	
☒	☐	Antibodies
☐	☒	Eukaryotic cell lines
☒	☐	Palaeontology and archaeology
☒	☐	Animals and other organisms
☒	☐	Human research participants
☒	☐	Clinical data
☒	☐	Dual use research of concern

Methods

n/a	Involved in the study	
☒	☐	ChIP-seq
☒	☐	Flow cytometry
☒	☐	MRI-based neuroimaging

Eukaryotic cell lines

Policy information about cell lines

Cell line source(s)	TCC – Trypanosomatid Culture Collection, Department of Parasitology, University of São Paulo, Avenida Lineu prestes 1374, Butanta, Sao Paulo, Brazil.
Authentication	Two representative strains for the six T. cruzi DTUs (TcI-TcVI), one from Tcbat (TcVII), T. cruzi marinkellei, Trypanosoma dionisii, Trypanosoma erneyi and Trypanosoma rangeli (Table 1) were comparatively analyzed in this study. These parasites have been previously characterized using genetic markers35,75.
Mycoplasma contamination	All cell lines were tested for mycoplasma contamination.
Commonly misidentified lines (See ICLAC register)	All cell lines used in this study were accurately identified.
